# Oxidative Stress and Cognitive Alterations Induced by Cancer Chemotherapy Drugs: A Scoping Review

**DOI:** 10.3390/antiox10071116

**Published:** 2021-07-13

**Authors:** Omar Cauli

**Affiliations:** 1 Frailty and Cognitive Impairment Group (FROG), University of Valencia, 46010 Valencia, Spain; Omar.Cauli@uv.es; Tel.: +34-96-386-41-82; Fax: +34-96-398-30-35; 2 Department of Nursing, University of Valencia, 46010 Valencia, Spain

**Keywords:** taxanes, anthracyclines, platinum, cognition, biomarker, *N-*acetylcysteine, clinical trial

## Abstract

Cognitive impairment is one of the most deleterious effects of chemotherapy treatment in cancer patients, and this problem sometimes remains even after chemotherapy ends. Common classes of chemotherapy-based regimens such as anthracyclines, taxanes, and platinum derivatives can induce both oxidative stress in the blood and in the brain, and these effects can be reproduced in neuronal and glia cell cultures. In rodent models, both the acute and repeated administration of doxorubicin or adriamycin (anthracyclines) or cisplatin impairs cognitive functions, as shown by their diminished performance in different learning and memory behavioural tasks. Administration of compounds with strong antioxidant effects such as *N-*acetylcysteine, gamma-glutamyl cysteine ethyl ester, polydatin, caffeic acid phenethyl ester, and 2-mercaptoethane sulfonate sodium (MESNA) counteract both oxidative stress and cognitive alterations induced by chemotherapeutic drugs. These antioxidant molecules provide the scientific basis to design clinical trials in patients with the aim of reducing the oxidative stress and cognitive alterations, among other probable central nervous system changes, elicited by chemotherapy in cancer patients. In particular, *N-*acetylcysteine and MESNA are currently used in clinical settings and are therefore attracting scientific attention.

## 1. Introduction

The study of cancer treatments currently occupies a prominent place in research and public health policies. These focus not only on curative or palliative purposes, but also on reducing the toxicity of cancer treatments which can, in turn, increase adherence to oncology protocols and improve quality of life and survival [1,2,3]. Antineoplastic therapies entail multiple side effects and must be closely monitored to promote better assimilation of the treatment as well as to encourage patient adherence to chemotherapy regimens and mitigate reductions in their quality of life. Chemotherapy is currently one of the most important tools in the fight against cancer; many of its side effects are well known, however, others such as cognitive impairment are still being studied. 

Cognitive impairment appears in up to 50–75% of people who undergo chemotherapy [4,5]. Some cancer patients report difficulties in concentration, memory, and attention both during and after the process of treating the disease, referred colloquially as ‘chemofog’ or ‘chemobrain’. Although in most cases the damage may be subtle and temporary, in a subgroup of patients, these alterations are more severe and can persist for years [6,7]. However, it should be borne in mind that even a subtle cognitive deterioration can have substantial repercussions on daily life [8,9]. Several factors such as the combination of cancer treatments, dose used, administration route, previous genetic vulnerability, and some psycho-social characteristics, among others, could give rise to these individual differences. However, chemotherapy drugs such as doxorubicin, cisplatin, 5-fluorouracil, methotrexate, and other anti-neoplastic agents trigger cognitive dysfunction in many patients [10,11,12,13,14]. These drugs are widely used in cancer chemotherapy to treat many cancers, including lymphoma, sarcoma, breast cancer, and many pediatric cancers [15,16]. About one third of women with breast cancer and half of children with cancer are treated with anthracyclines, including doxorubicin and epirubicin, and the class of taxanes, which includes paclitaxel among other drugs that are commonly used [17,18].

Oxidative stress is a dynamic and complex condition characterised by an imbalance between the generation of reactive oxygen species (ROS) and the availability and action of antioxidants [19,20,21]. The central nervous system consumes large amounts of oxygen to carry out physiological processes, leading to elevated free radical generation [22,23]. Some factors make the CNS susceptible to ROS attack, such as the deficit of antioxidant mechanisms, presence of high levels of polyunsaturated fatty acids, and selectivity of the blood-brain barrier, which reduces the diffusion of some antioxidants [23,24]. The escape of ROS from antioxidant mechanisms and their progressive accumulation trigger lipid peroxidation mechanisms as well as structural damage to proteins and DNA [25].

Oxidative stress is a factor in several neurodegenerative diseases, including Alzheimer’s disease, Parkinson’s disease, and amyotrophic lateral sclerosis [26,27,28] and is also thought to be a cornerstone of the pathophysiological mechanisms of drug-induced damage in several organs and tissues [29,30,31,32]. Indeed, several reports have shown that several pathophysiological factors lead to cognitive impairment after chemotherapy administration [33,34,35,36]. For instance, an increase in inflammatory markers in blood leading to their entry into the brain, white matter alterations, impaired neurogenesis, and cerebrovascular alterations have all have been proposed as possible factors that contribute to cognitive impairment. Moreover, both in vivo data obtained in animal models and in vitro experiments in cultured cells suggest that chemotherapy produces both an increase in oxidative stress and a decrease in antioxidative enzymes.

This knowledge has encouraged research designed to identify the role of chemotherapy-related oxidative stress as a mechanism of neurotoxicity. Thus, potential antioxidant treatments have been tested in preclinical research and, more recently, in studies in humans. Furthermore, studies in laboratory animals are facilitating the analysis of the efficacy of neuroprotective drugs to prevent the cognitive alterations caused by antineoplastic therapies. Although cognitive impairment induced by chemotherapy may resolve or improve once treatment is completed, it may often persist for decades or longer after treatment. In addition, despite the high frequency of cognitive impairment in cancer patients and survivors, effective treatments for this dysfunction are still lacking. Thus, this review focuses on the effects of chemotherapeutic drugs on oxidative stress in brain cells in vitro as well as in animal models and humans. We also assessed the type of molecules involved in chemotherapy-induced oxidative stress and potential treatments to prevent the oxidative stress and cognitive dysfunction associated with chemotherapeutic drugs.

## 2. Materials and Methods

**Figure 1:**
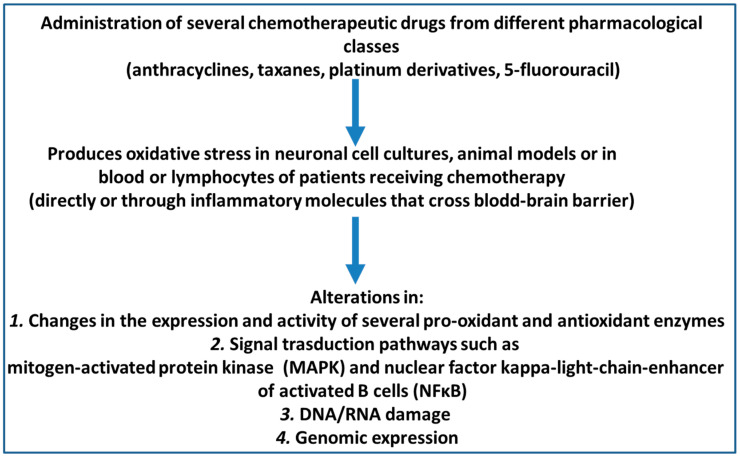
Oxidative stress mechanisms related to cognitive impairment after chemotherapy administration.

The objective of this review was to analyse original research articles published about the effect of oxidative stress and cognitive function outcomes after the application of chemotherapy agents in animal models and patients with cancer. In addition, the effects of chemotherapy agents on brain cell cultures were also reviewed. To that end, we analysed every original article in the PubMed/Medline and Cochrane Library electronic bibliographic databases that had been published up until March 2020 and that met the following inclusion criteria: (1) availability of the full text in English, Spanish, or Portuguese; (2) being a primary research article; and (3) presentation of identifiable data measuring oxidative stress and cognitive functions after the use of chemotherapy drugs in animal or human studies. To determine whether these articles fulfilled the inclusion criteria, I retrieved and checked the full text of these articles. Finally, the reference lists of all the relevant articles were manually cross-referenced to identify any additional articles of relevance. The database searches were performed according to PRISMA guidelines [37]. The primary search terms used were the MESH terms “cognition” AND one of the terms “chemotherapy AND cancer”, “anthracyclines”, “taxanes”, or “oncology”. A total of 272 documents resulted for the PubMed/Medline searches: “oxidative stress and cognition and chemotherapy and cancer”: 165; “oxidative stress and cognition and anthracyclines”: 25; “oxidative stress and cognition and taxanes”: 2; and “oxidative stress and cognition and oncology”: 180. The 10 document results for Cochrane Library were: “oxidative stress and cognition and chemotherapy and cancer”: 9 “oxidative stress and cognition and anthracyclines”: 0 “oxidative stress and cognition and taxanes”: 0 “oxidative stress and cognition and oncology”: 1. The full text was retrieved if data measuring oxidative stress and cognitive functions after the use of chemotherapy drugs in animal or human studies appeared in the abstract. The results were analysed separately for in vitro data collection performed in neuronal and glia cell cultures, animals s, and humans. The main findings are schematized in Figure 1.

## 3. Neuronal and Glial Oxidative Stress Induced by Chemotherapeutic Drugs in Preclinical Studies

Compared to in vivo studies, relatively few in vitro studies have investigated the direct effects of anticancer drugs on neurons [38,39,40]. In primary cultures of rat neural stem cells or progenitor cells and hippocampal neurons, cisplatin and temozolomide induced mitochondrial DNA damage, impaired respiratory activity, and increased oxidative stress [39,40]. However, the presence of antioxidants in the culture medium used in cell culture experiments may be sufficient to block the effect of anticancer drugs and therefore, these systems are not suitable for examining ROS-mediated neurotoxicity because they may not reflect the actual conditions of chemotherapy-treated animal models or patients.

Thus, to mimic the in vivo conditions of chemotherapy, an elegant study showed that methotrexate, 5-fluorouracil, or cisplatin neurotoxicity only occurred when primary cell cultures obtained from the cortical and striatal neurons from rat embryos were incubated with low concentrations of antioxidant substances [38,41]. Furthermore, the co-administration of methotrexate and 5-fluorouracil through incubation, as assessed with a cell-permeable fluorogenic probe (DHR123), showed a significant increase in intra-mitochondrial ROS. Together, these data indicate that oxidative stress plays a fundamental role in the mediation of in vitro neuronal toxicity. In other work, a far-red photostable fluorogenic probe (CellROX Deep Red Reagent) was used in primary cultures of rat hippocampal neurons to show that ROS was not increased by cyclophosphamide but that exposure to doxorubicin led to a 3-fold increase in CellROX signal intensities [42]. These data suggest that neurotoxicity is drug-dependent, and the main mechanism of chemotherapy-related cognitive impairment is unlikely to be increased oxidative stress.

In addition to mitochondria, peroxisomes also generate ROS, which in turn, promote cell senescence [43,44]. These ubiquitous cytoplasmic organelles are single-membrane vesicles that are found in most eukaryotic cells [45]. Peroxisomes are oxidative organelles in which molecular oxygen acts as a co-substrate for the formation of hydrogen peroxide. Of note, the anthracycline derivative, doxorubicin, affects peroxisomal homeostasis in neurons [46]. Moreover, in an H_2_O_2_ environment, the level of oxidative stress was enhanced in neurons [47] and primary cultured doxorubicin-treated neurons from rat embryos displayed an increase in oxidative stress in peroxisomes [48]. Besides neurons, some chemotherapeutic drugs have been shown to promote oxidative stress in glia cells [49]. In cultures of primary rat astrocytes, oxaliplatin induced an increase in superoxide anion production up to 10-fold compared to the controls and also induced the oxidation of lipids, proteins, and DNA [49,50]. Indeed, the level of protein carbonylation was approximately doubled in oxaliplatin-treated cells compared to control samples. Furthermore, in astrocyte cultures, the basal concentration of the oxidative stress marker 8-OH-dG also increased up to 9-fold after incubation with oxaliplatin [49]. Oxidative stress is often reported to be accompanied by DNA damage. For instance in primary neurons, the anthracycline doxorubicin, promotes the formation of DNA double-strand breaks and reduced synaptic and neurite density [51].

## 4. In Vivo Studies in Animal Models

Induction of oxidative stress after the administration of some chemotherapeutic drugs has also been demonstrated in experimental animals [38,52]. For example, the delivery of acute intraperitoneal doses of doxorubicin (25 mg/kg) in mice increased the concentration of protein carbonyl content and protein-bound 4-hydroxy-2-nonenal levels in the brain (as proxies for protein and lipid damage, respectively) [53]. Administration of another drug in the anthracycline family, adriamycin (20 mg/kg body weight) decreased the concentration of the antioxidant glutathione and reduced/oxidised glutathione ratio, increased the levels of the pro-oxidant enzyme glutathione peroxidase, and reduced the levels of the glutathione-S-transferase and glutathione reductase antioxidant enzymes [52]. Furthermore, the combination of doxorubicin and cyclophosphamide (intraperitoneally injected once a week for 2 weeks) reduced the glutathione and glutathione disulfide ratios in rat hippocampus tissue, a key brain structure in learning and memory processes [54].

The oxidative stress effects of anthracyclines in animal models appear to be mediated by the production of inflammatory cytokines. For example, the administration of anthracyclines increases blood TNF-alpha concentrations which, in turn, cross the blood-brain barrier and lead to brain alterations [53,55]. Confirming this, anthracyclines have a reduced effect on the brains of TNF alpha knock-out mice [55]. In other work, Bagnall-Moreau et al. [42] evaluated the effects of doxorubicin and cyclophosphamide (a combination referred to as AC) in a surgically ovariectomised rats and evaluated the mRNA expression of the antioxidant and oxidative-stress responsive genes in the hippocampus. They found that AC treatment increased the levels of several proteins involved in oxidative stress such as glutathione peroxidase (an enzyme whose main function is to protect against the degrading effect of endogenous hydroperoxides); the nuclear factor kappa-light-chain-enhancer of activated B cells (NFκB) p65 subunit (a protein complex that controls DNA transcription, including the expression of antioxidant enzymes); and peroxiredoxin-1 (an antioxidant enzyme that reduces the formation of hydrogen peroxide and alkyl hydroperoxides).

In contrast, the brain levels of heme-oxygenase 1, a cytoprotective enzyme that responds to oxidative and/or inflammatory stimuli, decreased in AC-treated animals [42]. Systemic AC chemotherapy also induced oxidative modifications to nucleic acids in the brain, shown as increases the levels of the DNA/RNA oxidation marker, 8-hydroxy-2’-deoxyguanosine (8–OH (d)G) and the level of oxidised 28S ribosomal RNA bands in the hippocampus of AC-chemotherapy treated rats [42]. Doxorubicin administration (once a week for four weeks) in rats impaired spatial learning and memory tasks and was accompanied by alterations in oxidative stress markers in the hippocampus and prefrontal cortex-brain structures that are key to learning and memory processes [56,57]. These effects appeared to be mediated by increased TNF-alpha, PGE2, and COX-2 concentrations, which in turn, produced an increase in malondialdehyde (a lipid peroxidation marker) and a reduction in reduced glutathione in the hippocampal tissue [56,57] and in the prefrontal cortex [57].

Chemotherapy-induced oxidative stress can damage DNA leading to genomic alterations in the DNA and/or RNA damage can be a direct consequence of oxidative stress. Administration of AC chemotherapy induces oxidative modifications to nucleic acids in the brain, as immuno-Northern blotting reveals 8–OH (d)G adducts in total RNA content and higher levels of oxidized 28S ribosomal RNA in the hippocampus of AC-treated rats [58].

Administration of mitomycin C, an antitumor antibiotic, in mice induces oxidative DNA damage in the prefrontal cortex, evidenced by the accumulation of 8-oxo-2’-deoxyguanosine (8-oxodG) and a decrease in the level of the 8-oxodG repair enzyme 8-oxoguanine glycosylase, a DNA glycosylase enzyme involved in base excision repair [59] in the prefrontal cortex of female animals 3 weeks after drug treatment [60]. In addition this, this drug reduced global DNA methylation and increased DNA hydroxymethylation in the prefrontal cortex of female mice [60]. Administration of doxorubicin-cyclophosphamide chemotherapy in a xenograft mice model of triple-negative breast cancer induces DNA damage as measured by the phosphorylation of the Ser-139 residue of the histone variant H2AX [61], forming γH2AX, an early cellular response to the induction of DNA double-strand breaks. Detection of this phosphorylation event is considered an highly specific and sensitive molecular marker for monitoring DNA damage [62]. Administration of the chemotherapeutic drug 5-fluorouracil in mice altered DNA structures in the brain as measured by an alteration of the amount of single- and double-strand DNA breaks as seen with a single cell gel electrophoresis assay (the Comet assay) [48].

The signal transduction pathways associated with oxidative stress after chemotherapy administration has been investigated by Bagnall-Moreau and coworkers after AC administration [42] at the level of mitogen-activated protein kinase (MAPK) cascades, which are involved in a number of stress response signalling pathways in the brain [63,64]. The phosphorylated levels of ERK/MAPK were found to be significantly higher in hippocampal lysates from AC chemotherapy treated rats. To examine pathway specificity, the activation of the ERK/MAPK and SAPK/JNK pathways and their (direct) substrates p90 RSK and c-Jun were measured after AC administration. Significantly higher levels of activated (phosphorylated) SAPK/JNK (Thr183/Tyr185) were detected in the hippocampus of AC-treated rats, and furthermore, the phosphorylated levels of a downstream target of the JNK pathway, c-Jun and the phosphorylation of ERK 1/2 were also found to be increased after AC treatment. In contrast, no changes were reported in the same brain area regarding p90 RSK. Thus, it appears that a global oxidative stress response is mounted on AC treatment, and it is reflected in the upregulation of multiple MAPK pathways [42,58].

## 5. Lipid Peroxidation

It is well established that oxidative stress promotes lipid peroxidation and has not only been associated with cancer itself but also with the mechanism of action of the cytostatic drugs used in chemotherapy in oncological patients [65,66,67,68]. A crucial mediator of lipid peroxidation is 4-hydroxynonenal (4-HNE), a by-product mainly derived from the peroxidation of omega-6 fatty acids [69]. The biological effects of 4-HNE are mainly due to the covalent modification of important biomolecules, such as proteins, DNA, and phospholipids containing an amino group [70]. HNE has a strong binding affinity for proteins and can therefore diffuse from the site where it has been formed to more distant sites in the body, and thus it can influence the proliferation, differentiation, and apoptosis of cancer cells on the one hand, while on the other hand, it can also affect the functionality of the genome [66,71,72,73].

Recently, studies have emerged suggesting that neurocognitive deficits that manifest before drug treatment may be associated with the presence of the tumor, a phenomenon recently termed ‘tumor brain’ [74]. To dissect the molecular mechanisms of tumor brain, the best experimental model is the TumorGraftTM obtained by grafting part of a patient’s tumor into immunodeficient mice [75]. Increased oxidative lipid damage (elevated levels of 4-hydroxy-2-nonenal) is observed in the hippocampal tissues of mice bearing triple-negative (TNBC) or progesterone receptor-positive (PR + BC) xenografts. In addition, TNBC and PR + BC tumor growth altered global gene expression in the hippocampus and affected multiple pathways [76]. An alteration of PI3K-Akt and MAPK signaling as well as other pathways crucial for the proper functioning of hippocampal neurons and a decrease in levels of the neuronal transcription factor NPAS4 [76], a regulator that governs the expression of brain-derived neurotrophic factor, a key brain neurotrophic factor crucial for cognitive functions [74,77,78]. The deleterious effects of cranial irradiation therapy, a technique widely used for cancer treatment, are accompanied by lipid peroxidation in the brain. In mice, cranial irradiation modifies the population of immature and proliferating neurons in the hippocampus, which is accompanied by an increase in 4-HNE-positive cells in various parts of the hippocampus (subventricular zone, granule cell layer, and hilum) [79]. Interestingly, administration of melatonin, a potent antioxidant, decreases HNE expression in the hippocampus, suggesting the possible benefit of melatonin treatment to combat cranial radiotherapy-induced cognitive alterations in cancer patients [79].

In cancer patients, administration of doxorubicin in the context of multi-agent chemotherapy regimens increases the concentration of HNE bound to plasma proteins [80,81]. Interestingly, in patients treated with the antioxidant drug MESNA, used to reduce the risk of hemorrhagic cystitis in patients receiving a chemotherapy drug of the oxazaphosphorine family, no increase in blood 4-NHE is observed. This has been also confirmed in animal models, in which MESNA administration antagonizes the doxorubicin-induced increase in plasma oxidative stress indexed by protein carbonyls and protein-bound HNE [82].

However, 4-HNE also has a physiological and protective role as a signalling molecule that stimulates gene expression and cell survival [83] and also has the potential to regulate blood–brain barrier function and thus interfere with the passage of chemotherapeutic drugs from the blood to the brain [72,84].

4-HNE also plays a cytotoxic role by inhibiting gene expression and promoting cell death, whereby chemotherapeutic drugs may exert their beneficial effects through oxidative stress. Confirming these protective functions, this molecule is of great importance for the anticancer effects of several anticancer therapies besides radiotherapy as well as cisplatin, cyclophosphamide, doxorubicin [69,73,85]. Indeed, all antineoplastic agents generate some ROS by inducing apoptosis in cancer cells [86,87], and an adequate level of oxidative stress interferes with the cellular processes that are necessary for antineoplastic agents to exert their optimal cytotoxicity on cancer cells, and modest levels of oxidative stress have been shown to reduce the cytotoxicity of anticancer drugs.

## 6. Treatment to Prevent Oxidative Stress and Cognitive Dysfunction Induced In Vivo by Chemotherapeutic Drugs

Different in vitro studies in neuronal and glia cell cultures have demonstrated that the oxidative stress induced by exposure to the chemotherapeutic drugs used in cancer treatment is reduced by molecules with antioxidant properties, as summarised in Table 1 [49,50,53,82,85]. However, treatment with acetyl-carnitine, which has been shown to decrease the ROS formation induced by doxorubicin exposure to neurons in vitro [58], has not been tested in vivo in animal models. Co-administration of different antioxidant compounds in animal models reduced oxidative stress levels and improved the cognitive deficits elicited by the administration of chemotherapeutic drugs [52,53,54,56,57,88,89].

*N-*acetylcysteine treatment (250 mg/kg/day) prevented free radical production, ameliorated apoptotic cellular death and dendritic spine loss, and partially reversed cisplatin-induced cognitive impairments [39]. A regimen of repeated cisplatin treatment in rats led to impaired cognitive performance (contextual fear conditioning, context object discrimination, and novel object recognition tasks), but this effect was partially mitigated by concomitant *N-*acetylcysteine treatment [88]. Moreover, administration of gamma-glutamyl cysteine ethyl ester, a glutathione precursor (150 mg/kg) prior to adriamycin administration (20 mg/kg body weight) led to a decreased production of protein oxidation and lipid peroxidation [89]. Furthermore, *N-*acetylcysteine reversed the anxiety-like behaviour and recognition memory task inhibition induced by doxorubicin and cyclophosphamide in rats [54]; this effect was also accompanied by a parallel improvement in the rats’ hippocampal GSH/GSSG ratios [54].

Polydatin, a resveratrol glycoside and potent natural antioxidant [90] extracted from the root of *Polygonum cuspidatum,* also counteracted the effect of the anthracycline drug doxorubicin. Prior treatment with polydatin inhibited doxorubicin-induced cognitive deficits in rats, both at the neurobehavioral and hippocampal histopathological levels [56]. Administration of caffeic acid phenethyl ester, a natural polyphenolic compound that exhibits unique context-dependent antioxidant activity was also able to counteract behavioural impairment and oxidative stress in hippocampal and prefrontal cortical tissues in rats treated with doxorubicin, as measured by the reduced glutathione content and malondialdehyde concentration in these brain areas [57].

Finally, administration of the antioxidant drug 2-mercaptoethane sulfonate sodium (MESNA) improved the production of oxidative stress markers in the blood and brains of rats treated with doxorubicin (determined using protein carbonyl and protein-bound 4-hydroxy-2-nonenal as indicators of protein oxidation and lipid peroxidation, respectively) [53,82]. In parallel, MESNA administration prevented the memory deficits induced by doxorubicin in the object recognition task [53]. These latter results are very promising for future clinical trials because MESNA is already being used in oncology patients to prevent urothelial toxicity including haemorrhagic cystitis, microhaematuria, and macrohaematuria in patients treated with chemotherapeutic drugs belonging to the oxazaphosphorine family (ifosfamide and cyclophosphamide) at doses considered to be urotoxic [91,92].

## 7. Oxidative Stress Markers After Chemotherapy Administration in Cancer Patients

In lung cancer patients, the concentration of the oxidative DNA damage markers 8-oxoguanine (8-oxoGua) and levels of 8-oxo-2’-deoxyguanosine (8-oxodG) in urine and whole blood were higher than in controls [93]. In addition, patients with stage IV cancer had higher urinary 8-oxoGua and 8-oxodG levels than patients with stage I–III disease. These results suggest that cancer promotes oxidative stress per se [94,95,96] and so, oxidative stress markers should be measured before chemotherapy administration in order to assess the effects of chemotherapeutic drugs versus those of the cancer alone. Urinary 8-oxodG levels have been shown to increase after radiotherapy and after six cycles of chemotherapy in lung cancer. Moreover, DNA oxidation parameters were increased both after radiotherapy and chemotherapy, suggesting that a pathophysiological mechanism such as the anti-cancer effects of these drugs may underlie these effects [93].

In previously untreated cancer patients (mainly with breast or endometrial tumours), Cadeddu et al. [97] evaluated various effects of the anthracycline drug epirubicin, including oxidative stress markers [97]. The levels of ROS were determined in blood samples using the free oxygen radicals test (FORT, with 1 FORT-U corresponding to the oxidative stress elicited by 0.26 mg/L of H_2_O_2_) [98]. In addition, they also measured the antioxidant enzyme glutathione peroxidase in red blood cells [97]. The administration of epirubicin promoted ROS formation and reduced the expression of glutathione peroxidase, suggesting that this drug induced oxidative stress.

Cisplatin-induced toxicities mainly seem to be caused by the formation of free radicals, leading to oxidative organ damage [99,100]. In fact, the plasma concentrations of the antioxidants vitamin C, E, and ceruloplasmin decreased after the administration of cisplatin or cisplatin-containing chemotherapy regimens. This appears to be a drug-induced effect because the concentrations of these substances returned to their initial levels just before the start of the next chemotherapy cycle. In addition, the levels of the antioxidants bilirubin and albumin also gradually decreased when measured just before the start of the next chemotherapy cycle. Furthermore, the copper/ceruloplasmin ratio, a marker of pro-oxidative status, significantly increased in the first cycle of cisplatin-based regimens [99]. In analogy of in vitro and in vivo studies as well as in human studies, direct DNA damage has been reported as a result of the mechanism of the action of chemotherapeutic drugs and oxidative stress. This has only been studied in lymphocytes derived from patients treated with chemotherapy. A substantial increase in both oxidative and direct DNA damage measured in the peripheral lymphocytes assessed by the Comet assay have been reported from before to shortly after chemotherapy administration in cancer patients [101,102,103,104,105].

## 8. Conclusions

Our review summarises preclinical evidence that several chemotherapeutic drugs widely used in cancer patients such as anthracyclines, taxanes, and platinum derivatives induced oxidative stress noted in the blood and brain, which may affect both neurons and glia cells. In animal models, the oxidative stress induced by chemotherapeutic drugs is accompanied by cognitive deficits. Administration of several antioxidants decreased or prevented these effects and helped pinpoint the potential role of antioxidants as drugs that may be able to reduce both oxidative stress and cognitive dysfunction caused by chemotherapy. Some recent trials performed in cancer patients suggested that *N-*acetylcysteine at doses of 1200 mg or 2400 mg/daily counteracted oxidative stress (expressed as an increase in serum malondialdehyde levels) and improved the incidence of peripheral neuropathy induced by paclitaxel [106]. Because *N-*acetylcysteine can also cross the blood–brain barrier [107,108], it may be a useful means of protection from the cognitive impairment induced by paclitaxel or taxanes, and perhaps by other chemotherapy drugs as well. This could be applied, for example, in women with breast cancer, in which the prior administration of chemotherapy, especially with taxanes, increased the odds of the appearance of more cognitive complaints [52]. Future trials with antioxidants should aim to evaluate use compliance and/or inadequate supplementation with *N-*acetylcysteine to compare the efficacy of these treatments with previous studies [100].

## Figures and Tables

**Table 1:** Drugs proven to decrease both the oxidative stress and cognitive dysfunction induced by the administration of chemotherapeutic drugs in animal models.

Antioxidant Compound	Drug-Induced Cognitive Impairment and Oxidative Stress	Behavioural Test Used to Assess Cognitive Function
*N-*acetylcysteine [88]		Recognition memory task
	Cisplatin	Fear conditioning learning
		Object discrimination
*N-*acetylcysteine [54] Doxorubicin Recognition memory task Cyclophosphamide		
Gamma-glutamyl cysteine ethyl ester [89]	Adriamycin	Recognition memory task
	
Polydatin [56]		Morris water-maze task
	Doxorubicin	Step-down avoidance task
	
	
Caffeic acid phenethyl ester [57]	Doxorubicin	Passive avoidance test
		Morris water-maze task
MESNA [53]	Doxorubicin	Recognition memory task

MESNA, 2-mercaptoethane sulfonate sodium.

## References

[1] Janelsins M.C., Kesler S.R., Ahles T.A., Morrow G.R. (2014). Prevalence, mechanisms, and management of cancer-related cognitive impairment. Int. Rev. Psychiatry.

[2] Matikas A., Foukakis T., Bergh J. (2017). Dose intense, dose dense and tailored dose adjuvant chemotherapy for early breast cancer: An evolution of concepts. Acta Oncol..

[3] Balducci L., Phillips D.M., Wallace C., Hardy C. (1987). Cancer chemotherapy in the elderly. Am. Fam. Physician.

[4] Ahles T.A., Saykin A.J., McDonald B.C., Li Y., Furstenberg C.T., Hanscom B.S., Mulrooney T.J., Schwartz G.N., Kaufman P.A. (2010). Longitudinal assessment of cognitive changes associated with adjuvant treatment for breast cancer: Impact of age and cognitive reserve. J. Clin. Oncol..

[5] Wefel J.S., Kesler S.R., Noll K.R., Schagen S.B. (2015). Clinical characteristics, pathophysiology, and management of noncentral nervous system cancer-related cognitive impairment in adults. CA. Cancer J. Clin..

[6] Miao H., Li J., Hu S., He X., Partridge S.C., Ren J., Bian Y., Yu Y., Qiu B. (2016). Long-term cognitive impairment of breast cancer patients after chemotherapy: A functional MRI study. Eur. J. Radiol..

[7] Christie L.A., Acharya M.M., Parihar V.K., Nguyen A., Martirosian V., Limoli C.L. (2012). Impaired cognitive function and hippocampal neurogenesis following cancer chemotherapy. Clin Cancer Res..

[8] Fehlauer F., Tribius S., Mehnert A., Rades D. (2005). Health-related quality of life in long term breast cancer survivors treated with breast conserving therapy: Impact of age at therapy. Breast Cancer Res. Treat..

[9] Walczak P., Janowski M. (2019). Chemobrain as a Product of Growing Success in Chemotherapy-Focus On Glia as Both a Victim and a Cure. Neuropsychiatry.

[10] Soussain C., Ricard D., Fike J.R., Mazeron J.J., Psimaras D., Delattre J.Y. (2009). CNS complications of radiotherapy and chemotherapy. Lancet.

[11] Winocur G., Berman H., Nguyen M., Binns M.A., Henkelman M., van Eede M., Piquette-Miller M., Sekeres M.J., Wojtowicz J.M., Yu J. (2018). Neurobiological Mechanisms of Chemotherapy-induced Cognitive Impairment in a Transgenic Model of Breast Cancer. Neuroscience.

[12] Joly F., Alibhai S.M.H., Galica J., Park A., Yi Q.L., Wagner L., Tannock I.F. (2006). Impact of Androgen Deprivation Therapy on Physical and Cognitive Function, as Well as Quality of Life of Patients With Nonmetastatic Prostate Cancer. J. Urol..

[13] Vardy J., Wefel J.S., Ahles T., Tannock I.F., Schagen S.B. (2008). Cancer and cancer-therapy related cognitive dysfunction: An international perspective from the Venice cognitive workshop. Ann. Oncol..

[14] Tannock I.F., Ahles T.A., Ganz P.A., van Dam F.S. (2004). Cognitive impairment associated with chemotherapy for cancer: Report of a workshop. J. Clin. Oncol..

[15] McGowan J.V., Chung R., Maulik A., Piotrowska I., Walker J.M., Yellon D.M. (2017). Anthracycline Chemotherapy and Cardiotoxicity. Cardiovasc. Drugs Ther..

[16] Tripaydonis A., Conyers R., Elliott D.A. (2019). Pediatric Anthracycline-Induced Cardiotoxicity: Mechanisms, Pharmacogenomics, and Pluripotent Stem-Cell Modeling. Clin. Pharmacol. Ther..

[17] Anampa J., Makower D., Sparano J.A. (2015). Progress in adjuvant chemotherapy for breast cancer: An overview. BMC Med..

[18] Jasra S., Anampa J. (2018). Anthracycline Use for Early Stage Breast Cancer in the Modern Era: A Review. Curr. Treat. Options Oncol..

[19] Costantini D. (2019). Understanding diversity in oxidative status and oxidative stress: The opportunities and challenges ahead. J. Exp. Biol..

[20] Ji L.L., Yeo D. (2021). Oxidative stress: An evolving definition. Fac. Rev..

[21] Jones D.P. (2006). Redefining oxidative stress. Antioxid. Redox Signal..

[22] Singh A., Kukreti R., Saso L., Kukreti S. (2019). Oxidative stress: A key modulator in neurodegenerative diseases. Molecules.

[23] Salim S. (2017). Oxidative stress and the central nervous system. J. Pharmacol. Exp. Ther..

[24] Franco R., Navarro G., Martínez-Pinilla E. (2019). Antioxidant Defense Mechanisms in Erythrocytes and in the Central Nervous System. Antioxidants.

[25] Poprac P., Jomova K., Simunkova M., Kollar V., Rhodes C.J., Valko M. (2017). Targeting Free Radicals in Oxidative Stress-Related Human Diseases. Trends Pharmacol. Sci..

[26] Radi E., Formichi P., Battisti C., Federico A. (2014). Apoptosis and oxidative stress in neurodegenerative diseases. J. Alzheimer’s Dis..

[27] Neves Carvalho A., Firuzi O., Joao Gama M., van Horssen J., Saso L. (2016). Oxidative Stress and Antioxidants in Neurological Diseases: Is There Still Hope?. Curr. Drug Targets.

[28] Ortiz G.G., Pacheco Moisés F.P., Mireles-Ramírez M., Flores-Alvarado L.J., González-Usigli H., Sánchez-González V.J., Sánchez-López A.L., Sánchez-Romero L., Díaz-Barba E.I., Santoscoy-Gutiérrez J.F. (2017). Oxidative Stress: Love and Hate History in Central Nervous System. Advances in Protein Chemistry and Structural Biology.

[29] Pereira C.V., Nadanaciva S., Oliveira P.J., Will Y. (2012). The contribution of oxidative stress to drug-induced organ toxicity and its detection in vitro and in vivo. Expert Opin. Drug Metab. Toxicol..

[30] Tafazoli S., Spehar D.D., O’Brien P.J. (2005). Oxidative stress mediated idiosyncratic drug toxicity. Drug Metab Rev..

[31] Martins M.R., Petronilho F.C., Gomes K.M., Dal-Pizzol F., Streck E.L., Quevedo J. (2008). Antipsychotic-induced oxidative stress in rat brain. Neurotox. Res..

[32] Kannarkat G., Lasher E.E., Schiff D. (2007). Neurologic complications of chemotherapy agents. Curr. Opin. Neurol..

[33] Mounier N.M., Abdel-Maged A.E.S., Wahdan S.A., Gad A.M., Azab S.S. (2020). Chemotherapy-induced cognitive impairment (CICI): An overview of etiology and pathogenesis. Life Sci..

[34] Ren X., Boriero D., Chaiswing L., Bondada S., St. Clair D.K., Butterfield D.A. (2019). Plausible biochemical mechanisms of chemotherapy-induced cognitive impairment (“chemobrain”), a condition that significantly impairs the quality of life of many cancer survivors. Biochim. Biophys. Acta-Mol. Basis Dis..

[35] Vitali M., Ripamonti C.I., Roila F., Proto C., Signorelli D., Imbimbo M., Corrao G., Brissa A., Rosaria G., de Braud F. (2017). Cognitive impairment and chemotherapy: A brief overview. Crit. Rev. Oncol. Hematol..

[36] Williams A.L.M., Shah R., Shayne M., Huston A.J., Krebs M., Murray N., Thompson B.D., Doyle K., Korotkin J., van Wijngaarden E. (2018). Associations between inflammatory markers and cognitive function in breast cancer patients receiving chemotherapy. J. Neuroimmunol..

[37] Rethlefsen M.L., Kirtley S., Waffenschmidt S., Ayala A.P., Moher D., Page M.J., Koffel J.B. (2021). PRISMA-S: An extension to the PRISMA Statement for Reporting Literature Searches in Systematic Reviews. Syst. Rev..

[38] Jiang Z.G., Fuller S.A., Ghanbari H.A. (2016). PAN-811 Blocks Chemotherapy Drug-Induced in Vitro Neurotoxicity, while Not Affecting Suppression of Cancer Cell Growth. Oxid. Med. Cell. Longev..

[39] Lomeli N., Di K., Pearre D.C., Chung T.F., Bota D.A. (2020). Mitochondrial-associated impairments of temozolomide on neural stem/progenitor cells and hippocampal neurons. Mitochondrion.

[40] Lomeli N., Lepe J., Gupta K., Bota D.A. (2021). Cognitive complications of cancer and cancer-related treatments—Novel paradigms. Neurosci Lett..

[41] Qian X., Li J., Xu S., Wan Y., Li Y., Jiang Y., Zhao H., Zhou Y., Liao J., Liu H. (2019). Prenatal exposure to phthalates and neurocognitive development in children at two years of age. Environ. Int..

[42] Bagnall-Moreau C., Chaudhry S., Salas-Ramirez K., Ahles T., Hubbard K. (2019). Chemotherapy-Induced Cognitive Impairment Is Associated with Increased Inflammation and Oxidative Damage in the Hippocampus. Mol. Neurobiol..

[43] Vallée A., Lecarpentier Y. (2018). Crosstalk between peroxisome proliferator-activated receptor gamma and the canonical WNT/β-catenin pathway in chronic inflammation and oxidative stress during carcinogenesis. Front. Immunol..

[44] Ganguli G., Mukherjee U., Sonawane A. (2019). Peroxisomes and oxidative stress: Their implications in the modulation of cellular immunity during mycobacterial infection. Front. Microbiol..

[45] Wilkinson C.F., Lamb IV J.C. (1999). The potential health effects of phthalate esters in children’s toys: A review and risk assessment. Regul. Toxicol. Pharmacol..

[46] Moruno-Manchon J.F., Uzor N.E., Kesler S.R., Wefel J.S., Townley D.M., Nagaraja A.S., Pradeep S., Mangala L.S., Sood A.K., Tsvetkov A.S. (2016). TFEB ameliorates the impairment of the autophagy-lysosome pathway in neurons induced by doxorubicin. Aging.

[47] Walker C.L., Pomatto L.C.D., Tripathi D.N., Davies K.J.A. (2018). Redox regulation of homeostasis and proteostasis in peroxisomes. Physiol. Rev..

[48] Moruno-Manchon J.F., Uzor N.E., Kesler S.R., Wefel J.S., Townley D.M., Nagaraja A.S., Pradeep S., Mangala L.S., Sood A.K., Tsvetkov A.S. (2018). Peroxisomes contribute to oxidative stress in neurons during doxorubicin-based chemotherapy. Mol. Cell. Neurosci..

[49] Di Cesare Mannelli L., Zanardelli M., Failli P., Ghelardini C. (2013). Oxaliplatin-induced oxidative stress in nervous system-derived cellular models: Could it correlate with in vivo neuropathy?. Free Radic. Biol. Med..

[50] Di Cesare Mannelli L., Zanardelli M., Landini I., Pacini A., Ghelardini C., Mini E., Bencini A., Valtancoli B., Failli P. (2016). Effect of the SOD mimetic MnL4 on in vitro and in vivo oxaliplatin toxicity: Possible aid in chemotherapy induced neuropathy. Free Radic. Biol. Med..

[51] Manchon J.F.M., Dabaghian Y., Uzor N.E., Kesler S.R., Wefel J.S., Tsvetkov A.S. (2016). Levetiracetam mitigates doxorubicin-induced DNA and synaptic damage in neurons. Sci. Rep..

[52] Joshi G., Aluise C.D., Cole M.P., Sultana R., Pierce W.M., Vore M., St Clair D.K., Butterfield D.A. (2010). Alterations in brain antioxidant enzymes and redox proteomic identification of oxidized brain proteins induced by the anti-cancer drug adriamycin: Implications for oxidative stress-mediated chemobrain. Neuroscience.

[53] Keeney J.T.R., Ren X., Warrier G., Noel T., Powell D.K., Brelsfoard J.M., Sultana R., Saatman K.E., St. Clair D.K., Butterfield D.A. (2018). Doxorubicin-induced elevated oxidative stress and neurochemical alterations in brain and cognitive decline: Protection by MESNA and insights into mechanisms of chemotherapy-induced cognitive impairment (“chemobrain”). Oncotarget.

[54] Kitamura Y., Ushio S., Sumiyoshi Y., Wada Y., Miyazaki I., Asanuma M., Sendo T. (2020). N-acetylcysteine attenuates the anxiety-like behavior and spatial cognition impairment induced by doxorubicin and cyclophosphamide combination treatment in rats. Pharmacology.

[55] Ren X., Keeney J.T.R., Miriyala S., Noel T., Powell D.K., Chaiswing L., Bondada S., St. Clair D.K., Butterfield D.A. (2019). The triangle of death of neurons: Oxidative damage, mitochondrial dysfunction, and loss of choline-containing biomolecules in brains of mice treated with doxorubicin. Advanced insights into mechanisms of chemotherapy induced cognitive impairment (“chemobr”). Free Radic. Biol. Med..

[56] Tong Y., Wang K., Sheng S., Cui J. (2020). Polydatin ameliorates chemotherapy-induced cognitive impairment (chemobrain) by inhibiting oxidative stress, inflammatory response, and apoptosis in rats. Biosci. Biotechnol. Biochem..

[57] Ali M.A., Menze E.T., Tadros M.G., Tolba M.F. (2020). Caffeic acid phenethyl ester counteracts doxorubicin-induced chemobrain in Sprague-Dawley rats: Emphasis on the modulation of oxidative stress and neuroinflammation. Neuropharmacology.

[58] Lee S., Rauch J., Kolch W. (2020). Targeting MAPK Signaling in Cancer: Mechanisms of Drug Resistance and Sensitivity. Int. J. Mol. Sci..

[59] Ba X., Boldogh I. (2018). 8-Oxoguanine DNA glycosylase 1: Beyond repair of the oxidatively modified base lesions. Redox Biol..

[60] Kovalchuk A., Rodriguez-Juarez R., Ilnytskyy Y., Byeon B., Shpyleva S., Melnyk S., Pogribny I., Kolb B., Kovalchuk O. (2016). Sex-specific effects of cytotoxic chemotherapy agents cyclophosphamide and mitomycin C on gene expression, oxidative DNA damage, and epigenetic alterations in the prefrontal cortex and hippocampus-An aging connection. Aging.

[61] Himmel L.E., Lustberg M.B., DeVries A.C., Poi M., Chen C.S., Kulp S.K. (2016). Minocycline, a putative neuroprotectant, co-administered with doxorubicin-cyclophosphamide chemotherapy in a xenograft model of triple-negative breast cancer. Exp. Toxicol. Pathol..

[62] Mah L.J., El-Osta A., Karagiannis T.C. (2010). γh2AX: A sensitive molecular marker of DNA damage and repair. Leukemia.

[63] Kaminska B., Gozdz A., Zawadzka M., Ellert-Miklaszewska A., Lipko M. (2009). MAPK signal transduction underlying brain inflammation and gliosis as therapeutic target. Anat. Record Anat Rec..

[64] Falcicchia C., Tozzi F., Arancio O., Watterson D.M., Origlia N. (2020). Involvement of p38 mapk in synaptic function and dysfunction. Int. J. Mol. Sci..

[65] Zhong H., Yin H. (2015). Role of lipid peroxidation derived 4-hydroxynonenal (4-HNE) in cancer: Focusing on mitochondria. Redox Biol..

[66] Gasparovic A.C., Milkovic L., Sunjic S.B., Zarkovic N. (2017). Cancer growth regulation by 4-hydroxynonenal. Free Radic. Biol. Med..

[67] Guéraud F. (2017). 4-Hydroxynonenal metabolites and adducts in pre-carcinogenic conditions and cancer. Free Radic. Biol. Med..

[68] Matsunaga T., Tsuchimura S., Azuma N., Endo S., Ichihara K., Ikari A. (2019). Caffeic acid phenethyl ester potentiates gastric cancer cell sensitivity to doxorubicin and cisplatin by decreasing proteasome function. Anticancer. Drugs.

[69] Dwivedi S., Sharma A., Patrick B., Sharma R., Awasthi Y.C. (2007). Role of 4-hydroxynonenal and its metabolites in signaling. Redox Rep..

[70] Gallo G., Sprovieri P., Martino G. (2020). 4-hydroxynonenal and oxidative stress in several organelles and its damaging effects on cell functions. J. Physiol. Pharmacol..

[71] Calonghi N., Boga C., Cappadone C., Pagnotta E., Bertucci C., Fiori J., Masotti L. (2002). Cytotoxic and cytostatic effects induced by 4-hydroxynonenal in human osteosarcoma cells. Biochem. Biophys. Res. Commun..

[72] Karlhuber G.M., Bauer H.C., Eckl P.M. (1997). Cytotoxic and genotoxic effects of 4-hydroxynonenal in cerebral endothelial cells. Mutat. Res. Fundam. Mol. Mech. Mutagen..

[73] Conklin K.A. (2004). Chemotherapy-associated oxidative stress: Impact on chemotherapeutic effectiveness. Integr. Cancer Ther..

[74] Lange M., Joly F., Vardy J., Ahles T., Dubois M., Tron L., Winocur G., De Ruiter M.B., Castel H. (2019). Cancer-related cognitive impairment: An update on state of the art, detection, and management strategies in cancer survivors. Ann. Oncol..

[75] Kovalchuk A., Ilnytskyy Y., Rodriguez-Juarez R., Shpyleva S., Melnyk S., Pogribny I., Katz A., Sidransky D., Kovalchuk O., Kolb B. (2017). Chemo brain or tumor brain–that is the question: The presence of extracranial tumors profoundly affects molecular processes in the prefrontal cortex of TumorGraft mice. Aging.

[76] Kovalchuk A., Ilnytskyy Y., Rodriguez-Juarez R., Katz A., Sidransky D., Kolb B., Kovalchuk O. (2018). Growth of triple negative and progesterone positive breast cancer causes oxidative stress and down-regulates neuroprotective transcription factor NPAS4 and NPAS4-regulated genes in hippocampal tissues of tumorgraft mice-An aging connection. Front. Genet..

[77] Navarro-Martínez R., Fernández-Garrido J., Buigues C., Torralba-Martínez E., Martinez-Martinez M., Verdejo Y., Mascarós M.C.M.C., Cauli O. (2015). Brain-derived neurotrophic factor correlates with functional and cognitive impairment in non-disabled older individuals. Exp. Gerontol..

[78] Deleemans J.M., Chleilat F., Reimer R.A., Henning J.W., Baydoun M., Piedalue K.A., McLennan A., Carlson L.E. (2019). The chemo-gut study: Investigating the long-term effects of chemotherapy on gut microbiota, metabolic, immune, psychological and cognitive parameters in young adult Cancer survivors; Study protocol. BMC Cancer.

[79] Manda K., Ueno M., Anzai K. (2009). Cranial irradiation-induced inhibition of neurogenesis in hippocampal dentate gyrus of adult mice: Attenuation by melatonin pretreatment. J. Pineal Res..

[80] Aluise C.D., Miriyala S., Noel T., Sultana R., Jungsuwadee P., Taylor T.J., Cai J., Pierce W.M., Vore M., Moscow J.A. (2011). 2-Mercaptoethane sulfonate prevents doxorubicin-induced plasma protein oxidation and TNF-α release: Implications for the reactive oxygen species-mediated mechanisms of chemobrain. Free Radic. Biol. Med..

[81] Aluise C.D., Sultana R., Tangpong J., Vore M., St Clair D., Moscow J.A., Butterfield D.A. (2010). Chemo brain (chemo fog) as a potential side effect of doxorubicin administration: Role of cytokine-induced, oxidative/nitrosative stress in cognitive dysfunction. Adv. Exp. Med. Biol..

[82] Aluise C.D., St Clair D., Vore M., Butterfield D.A. (2009). In vivo amelioration of adriamycin induced oxidative stress in plasma by gamma-glutamylcysteine ethyl ester (GCEE). Cancer Lett..

[83] Ayala A., Muñoz M.F., Argüelles S. (2014). Lipid peroxidation: Production, metabolism, and signaling mechanisms of malondialdehyde and 4-hydroxy-2-nonenal. Oxid. Med. Cell. Longev..

[84] Mertsch K., Blasig I., Grune T. (2001). 4-Hydroxynonenal impairs the permeability of an in vitro rat blood-brain barrier. Neurosci. Lett..

[85] Keeney J.T.R., Miriyala S., Noel T., Moscow J.A., St. Clair D.K., Butterfield D.A. (2015). Superoxide induces protein oxidation in plasma and TNF-α elevation in macrophage culture: Insights into mechanisms of neurotoxicity following doxorubicin chemotherapy. Cancer Lett..

[86] Cui Q., Wang J.Q., Assaraf Y.G., Ren L., Gupta P., Wei L., Ashby C.R., Yang D.H., Chen Z.S. (2018). Modulating ROS to overcome multidrug resistance in cancer. Drug Resist. Updates.

[87] Galadari S., Rahman A., Pallichankandy S., Thayyullathil F. (2017). Reactive oxygen species and cancer paradox: To promote or to suppress?. Free Radic. Biol. Med..

[88] Lomeli N., Di K., Czerniawski J., Guzowski J.F., Bota D.A. (2017). Cisplatin-induced mitochondrial dysfunction is associated with impaired cognitive function in rats. Free Radic. Biol. Med..

[89] Joshi G., Hardas S., Sultana R., St. Clair D.K., Vore M., Butterfield D.A. (2007). Glutathione elevation by γ-glutamyl cysteine ethyl ester as a potential therapeutic strategy for preventing oxidative stress in brain mediated by in vivo administration of adriamycin: Implication for chemobrain. J. Neurosci. Res..

[90] Du Q.H., Peng C., Zhang H. (2013). Polydatin: A review of pharmacology and pharmacokinetics. Pharm. Biol..

[91] Shaw I.C., Graham M.I. (1987). Mesna-a short review. Cancer Treat. Rev..

[92] Shaw I.C. (1987). Mesna and oxazaphosphorine cancer chemotherapy. Cancer Treat. Rev..

[93] Crohns M., Saarelainen S., Erhola M., Alho H., Kellokumpu-Lehtinen P. (2009). Impact of radiotherapy and chemotherapy on biomarkers of oxidative DNA damage in lung cancer patients. Clin. Biochem..

[94] Klaunig J.E. (2019). Oxidative Stress and Cancer. Curr. Pharm. Des..

[95] Reuter S., Gupta S.C., Chaturvedi M.M., Aggarwal B.B. (2010). Oxidative stress, inflammation, and cancer: How are they linked?. Free Radic. Biol. Med..

[96] Gill J.G., Piskounova E., Morrison S.J. (2016). Cancer, oxidative stress, and metastasis. Cold Spring Harb. Symp. Quant. Biol..

[97] Cadeddu C., Piras A., Mantovani G., Deidda M., Dessì M., Madeddu C., Massa E., Mercuro G. (2010). Protective effects of the angiotensin II receptor blocker telmisartan on epirubicin-induced inflammation, oxidative stress, and early ventricular impairment. Am. Heart J..

[98] Pavlatou M.G., Papastamataki M., Apostolakou F., Papassotiriou I., Tentolouris N. (2009). FORT and FORD: Two simple and rapid assays in the evaluation of oxidative stress in patients with type 2 diabetes mellitus. Metabolism.

[99] Weijl N.I., Elsendoorn T.J., Lentjes E.G.W.M., Hopman G.D., Wipkink-Bakker A., Zwinderman A.H., Cleton F.J., Osanto S. (2004). Supplementation with antioxidant micronutrients and chemotherapy-induced toxicity in cancer patients treated with cisplatin-based chemotherapy: A randomised, double-blind, placebo-controlled study. Eur. J. Cancer.

[100] Weijl N.I., Hopman G.D., Wipkink-Bakker A., Lentjes E.G.W.M., Berger H.M., Cleton F.J., Osanto S. (1998). Cisplatin combination chemotherapy induces a fall in plasma antioxidants of cancer patients. Ann. Oncol..

[101] Conroy S.K., McDonald B.C., Smith D.J., Moser L.R., West J.D., Kamendulis L.M., Klaunig J.E., Champion V.L., Unverzagt F.W., Saykin A.J. (2013). Alterations in brain structure and function in breast cancer survivors: Effect of post-chemotherapy interval and relation to oxidative DNA damage. Breast Cancer Res. Treat..

[102] Tomasello B., Malfa G.A., Strazzanti A., Gangi S., Di Giacomo C., Basile F., Renis M. (2017). Effects of physical activity on systemic oxidative/DNA status in breast cancer survivors. Oncol. Lett..

[103] Scuric Z., Carroll J.E., Bower J.E., Ramos-Perlberg S., Petersen L., Esquivel S., Hogan M., Chapman A.M., Irwin M.R., Breen E.C. (2017). Biomarkers of aging associated with past treatments in breast cancer survivors. NPJ Breast Cancer.

[104] Cruz-Jentoft A.J., Bahat G., Bauer J., Boirie Y., Bruyère O., Cederholm T., Cooper C., Landi F., Rolland Y., Sayer A.A. (2019). Sarcopenia: Revised European consensus on definition and diagnosis. Age Ageing.

[105] Root J.C., Pergolizzi D., Pan H., Orlow I., Passik S.D., Silbersweig D., Stern E., Ahles T.A. (2020). Prospective evaluation of functional brain activity and oxidative damage in breast cancer: Changes in task-induced deactivation during a working memory task. Brain Imaging Behav..

[106] Khalefa H.G., Shawki M.A., Aboelhassan R., El Wakeel L.M. (2020). Evaluation of the effect of N-acetylcysteine on the prevention and amelioration of paclitaxel-induced peripheral neuropathy in breast cancer patients: A randomized controlled study. Breast Cancer Res. Treat..

[107] Hara Y., McKeehan N., Dacks P.A., Fillit H.M. (2017). Evaluation of the Neuroprotective Potential of N-Acetylcysteine for Prevention and Treatment of Cognitive Aging and Dementia. J. Prev. Alzheimer’s Dis..

[108] Katz M., Won S.J., Park Y., Orr A., Jones D.P., Swanson R.A., Glass G.A. (2015). Cerebrospinal fluid concentrations of N-acetylcysteine after oral administration in Parkinson’s disease. Park. Relat. Disord..

